# Adult Attachment and Fear of Missing Out: Does the Mindful Attitude Matter?

**DOI:** 10.3390/healthcare11233093

**Published:** 2023-12-04

**Authors:** Matteo Perazzini, Danilo Bontempo, Marco Giancola, Simonetta D’Amico, Enrico Perilli

**Affiliations:** 1 Department of Life, Health and Environmental Sciences, University of L’Aquila, 67100 L’Aquila, Italy; matteo.perazzini@graduate.univaq.it (M.P.); danilo.bontempo@guest.univaq.it (D.B.); 2 Department of Biotechnological and Applied Clinical Sciences, University of L’Aquila, 67100 L’Aquila, Italy; marco.giancola@univaq.it (M.G.); simonetta.damico@univaq.it (S.D.)

**Keywords:** adult attachment, mindfulness, FoMO, young adults, social relationships, mediation

## Abstract

Fear of missing out (FoMO) involves the desire or urge to stay continuously connected to and kept up-to-date with a social reference group. The present study explored the relationships between adult attachment and FoMO and the potential mediating effect of a mindful attitude. The present study was carried out on 192 participants (mean_age_ = 23.24 years; SD_age_ = 4.33 years), of whom 151 (78.6%) were female and the remaining 41 (21.4%) were male. The participants completed the Adult Attachment Scale—Revised (AAS-R), which evaluates Close, Depend, and Anxiety attachment, the Cognitive and Affective Mindfulness Scale—Revised (CAMS-R) and the Fear of Missing Out Scale (FoMOs). The results indicated that the three forms of adult attachment (Close, Depend, and Anxiety) were associated with FoMO through the indirect effect of mindful attitudes. This study yielded relevant theoretical and practical implications regarding the critical role of a mindful attitude as a protective factor against FoMO. Limitations and future research directions were also discussed.

## 1. Introduction

Fear of missing out (FoMO) describes the fear of being excluded from or not participating in an experience [1]. Specifically, FoMO involves “a pervasive apprehension that others might be having rewarding experiences from which one is absent” [2] (p. 1841). It reflects people’s concerns, worries, and anxieties about being out of touch with satisfying events and experiences across their extended social circles. Given its nature, FoMO was found to be negatively implicated in social relationships, especially by social media and mobile devices [3]. Past research emphasized that the individual disposition toward FoMO elicits comparisons of another users’ lives to one’s own as portrayed by social media profiles and postings [4]. An upward social comparison often occurs due to the propensity to overestimate one’s positive experiences [5]. 

Furthermore, FoMO was found to be associated with negative emotional experiences such as anxiety, low self-esteem, depressive symptoms, negative physical symptoms, feelings of inadequacy, and low life satisfaction, as well as an increase in social envy and exclusion [2,6,7,8]. In this vein, individuals experiencing FoMO tend to spend more time comparing the ostensible lives of others to their own, and consequently, their own lives become less fulfilling if they replace face-to-face interactions with viewing other users’ profiles or recent posts to feel more connected or related to others [9]. 

Given this negative role in people’s lives, past research in the psychology field has sought to deepen the understanding of the main determinants of FoMO, especially in adolescents and young adults [10,11]. However, to our knowledge, little work has been done on how such determinants (i.e., intrapersonal factors) interact to shape FoMO. 

To fill this gap, the current research explored the joint contribution of adult attachment and mindful attitudes in shaping FoMO in a sample of young adults. Based on the idea that attachment triggers the mindful attitude that, in turn, affects the individual ability to manage affect, the current research advanced a mediation model in which adult attachment was entered as the independent variable, mindful attitude was the mediator, and FoMO was the dependent variable.

### Literature Review

Attachment theory has a long tradition in psychology literature. It entails an inborn and stable motivation system, which allows individuals to establish and maintain relationships with significant figures in their lives [12]. Attachment theory was first proposed by Bowlby [13] to define emotional bonds between children and their caregivers [14]. The experience of relationships with a significant adult (i.e., parents or caregivers) is crucial for children’s psychobiology, and the quality of these primary experiences affects the internalization of the attachment pattern with adaptive consequences on relationships during adulthood [15]. According to previous studies, adult attachment and social relationships (e.g., love relationships) in adulthood are derived from parental attachment [16]. Specifically, three main dimensions characterize adult attachment: (1) Close, which entails the degree to which the subject feels comfortable in establishing close relationships with their partner (thus, a higher level of Close demonstrates the individual tendency to establish intimacy with their partner); (2) Depend, which involves the individual’s ability to trust their partner when necessary; and (3) Anxiety, which refers to the individual’s fear and worry of being abandoned or rejected by their partner. Notably, these three dimensions can be associated with attachment style categories, providing information on how individuals form and establish their relationships. As shown by Collins and Read [17], high levels of Close and Depend attachment can be classified as a form of secure attachment. In addition, low levels of Close attachment and high levels of Anxiety and Depend attachment can be classified as the anxious attachment style. Finally, high levels of Anxiety and Depend attachment can be considered as a form of the avoidant attachment style. In past research, the AAS-R showed good psychometric properties [17].

Although the association between adult attachment and FoMO is not widely explored, some authors pointed out that subjects with an anxious attachment style tend to experience higher FoMO [18]. Indeed, individuals with attachment characterized by high levels of Anxiety to cope with their fear of abandonment show negative self-conception and attention-seeking and reassurance-seeking tendencies [19]. Similarly, people with an insecure attachment style experience anguish and fear when away from significant figures and experience higher levels of FoMO [20]. In turn, previous studies showed that secure attachment affects social relationships, which protects against feelings of social disconnection [21]. Indeed, people with secure attachment, given their adequate autonomy, high levels of self-efficacy and self-esteem, and the ability to have healthy relationships with others, are less likely to feel distant from others and experience FoMO [22]. Overall, these findings suggest that a higher ability to regulate anxiety and negative affect underpinned by secure attachment weakens the individual disposition to experience FoMO. In this vein, mindfulness can be considered as a critical factor in regulating emotions. Specifically, mindfulness involves “paying attention in a particular way: on purpose, in the present moment, and non-judgmentally” [23] (p. 4). This implies that mindfulness is a state in which the focus is on seeing and accepting things as they are without trying to change them [24]. Moreover, this mental state enables more attention to be given to situations and contexts, promoting an openness to points of view and new information by expanding mental capacities [25]. In addition to the state of mindfulness, the notion of a mindful attitude has also been advanced in the literature, having been characterized as a personality-based disposition that individuals may present toward mindfulness, which people present at different degrees regardless of mindfulness meditation or interventions [26]. Although some studies found inconsistent results, different authors stressed a close association between mindfulness and attachment. For instance, Walsh and colleagues [27] showed that low levels of anxious attachment negatively predicted mindfulness. Similarly, Shaver and colleagues [28] observed that avoidant attachment predicted the main facets of a mindful attitude, including describing, acting with awareness, nonjudging of experience, nonreactivity, and observing. Additionally, several studies [29,30] found that people with a secure attachment style reported higher mindfulness levels than insecurely attached individuals.

Interestingly, mindful attitudes were also found to be associated with FoMO [31,32,33]. Specifically, different authors pointed out that a mindful attitude weakens the effect of FoMO, enhancing happiness and wellbeing [34] as well as positive thinking and emotions [35]. Several studies confirmed this notion by demonstrating a reduction of FoMO through mindful practice [36]. For instance, Sofia and colleagues [36] suggested that some features of mindful practice (i.e., identifying body reactions and related emotions) contribute to reducing negative affect associated with FoMO with positive implications for individuals’ wellbeing [37]. Additionally, past research proved that a mindful attitude is a stable predictor of FoMO. Specifically, Jin and colleagues [38] found that people with a high degree of mindful attitude are less likely to experience FoMO through higher awareness of emotional states.

Drawing upon the literature mentioned above, the current research aimed to deepen the understanding of the main mechanisms underpinning FoMO. Specifically, we advanced a multidimensional perspective that involves adult attachment and a mindful attitude. Specifically, given that a mindful attitude originates from attachment experience and affects the individual disposition toward negative affect, such as FoMO, the main hypothesis of the present study was formulated as follows: a mindful attitude mediates the interplay between adult attachment and FoMO.

## 2. Materials and Methods

### 2.1. Participants and Procedure

Data were collected from January 2023 to July 2023 via an online survey using an online platform (Google Forms). The link was distributed through social media, including Instagram, Facebook, and WhatsApp. In addition, participants were recruited by word-of-mouth among students of different courses at the University of L’Aquila (psychology, sports science, medicine, biology, and biotechnology). In the current research, 192 participants were enrolled (mean_age_ = 23.24 years, SD_age_ = 4.33 years), of whom 151 (78.6%) were female and the remaining 41 (21.4%) were male. To participate in the study, informed consent was acquired from all participants. Through an online starting page, subjects were informed about the nature of the study and its main aims. Then, participants were shown a note detailing all measures adopted in the study. In addition, the note clearly explained that participation was anonymous, voluntary, and without any rewards. Afterwards, participants could begin the online survey consisting of two main sections. The first section involved a short socio-demographic questionnaire on age, gender, and area of residence. In addition, within this section, participants were asked through a single item if they had experience in mindfulness intervention: “Have you ever practiced mindfulness?”. In our sample, 38 participants (19.8%) declared mindfulness experience. In the second section, participants were requested to fill in the Fear of Missing Out Scale (FoMOS), the Adult Attachment Scale—Revised (AAS-R), and The Cognitive and Affective Mindfulness Scale—Revised (CAMS-R). The research was approved by the University Research Ethics Committee, in accordance with the Declaration of Helsinki.

### 2.2. Measures

*Fear of missing out.* The FoMO Scale (FoMOs; [2]) entails 10 items considering two main dimensions of FoMO: fear (e.g., “I fear my friends have more rewarding experiences than me”) and control (e.g., “It bothers me when I miss the opportunity to meet up with friends”). Participants were requested to answer based on a Likert-type scale ranging from 1 (not at all true of me) to 5 (extremely true of me). A total score was obtained by summing the scores from all 10 items, with higher scores indicating higher FoMO. In previous research, the FoMOs demonstrated good psychometric properties [39], and in the current study, the internal reliability was good (Cronbach’s α = 0.80).

*Adult attachment.* The Adult Attachment Scale—Revised (AAS-R; [16,40]) is a revised version of the original AAS [41] and assesses adult attachment with romantic partners. This instrument consists of 18 items on a 5-point Likert-type scale ranging from 1 (not at all characteristic of me) to 5 (very characteristic of me). The AAS-R enables the evaluation of the differences in adult attachment styles [41]. Specifically, the AAS-R evaluates three main dimensions of adult attachment, namely Anxiety (e.g., “I feel that others are reluctant to establish the intimacy with me that I would like to achieve with them”), Depend (e.g., “It seems that people are never there when you need them”), and Close (e.g., “Often, my partners would like to establish an emotional closeness beyond that which makes me feel comfortable”) in romantic situations. In the current research, the internal reliability ranges from acceptable to good. Cronbach’s α was as follows: Anxiety = 0.90, Depend = 0.67, and Close = 0.77. 

*Mindful attitude.* The Cognitive and Affective Mindfulness Scale—Revised (CAMS-R; [42]) comprises 12 items on a 4-point Likert-type scale from 1 (rarely/not at all) to 4 (almost always). The scale assesses: (1) the ability to regulate attention, which comprises the degree to which individuals experience their thoughts and feelings, considering attention—(e.g., “I can pay close attention to one thing for a long period”); (2) present focus, which describes the orientation to present experience (e.g., “I am able to focus on the present moment”); (3) awareness, which depicts the awareness of experience (e.g., “I can usually describe how I feel at the moment in considerable detail”); and (4) acceptance, which involves the attitude of nonjudgment towards experience (e.g., “I can accept the thoughts and feelings I have”) [43]. As in previous research, the scores of all items were summed, with higher scores reflecting a higher mindful attitude. Previous research demonstrated that the CAMS-R has acceptable psychometric properties [42]. The internal consistency across the 12 items was good (Cronbach α = 0.79).

*Confounding variables.* In this study, socio-demographics, including age, gender (0 = female; 1 = male), and education (years), were included as potential confounding variables. Given the aim of the current research, experience in mindfulness practice (0 = no; 1 = yes) was also collected. 

### 2.3. Statistical Analysis

Data were analyzed using the SPSS Statistics version 24 for Windows (IBM Corporation, Armonk, New York, NY, USA). Descriptive statistics and correlations were carried out to evaluate the main features and associations among all the study variables. The mediation analysis was performed using the PROCESS macro for SPSS (version 3.5) [44], selecting model 4. In PROCESS, the mediation effect is denoted by a significant 95% confidence interval (CI) bootstrapped based on 50,000 samples. As shown in previous research (e.g., [45,46]), the bootstrapping approach enables the computing of an accurate evaluation of the mediating and moderating effects in small- to medium-sized samples. The significance of the results is provided if the range of the bootstrapped CI does not include the value of zero [47]. All significance in this study was set to *p* < 0.05. 

## 3. Results

The normality test revealed that the variables of interest were normally distributed (Kolmogorov–Smirnov Test: Z_Adult Attachment-Close_ = 0.38, ns; Z_Adult attachment-Dependent_ = 0.30, ns; Z_Adult attchment-Anxiety_ = 0.31, sig; Z_FoMO_ = 0.20, ns; Z_Mindful attitude_ = 0.40, ns). No univariate outliers were found using the z-test, with −4.0 and +4.0 z-scores as the cutoff for samples larger than 100 [48,49]. Also, Harman’s single-factor test [50] indicated that the variance explained by a single-factor exploratory model was 29.36%, revealing no common method bias (CMB) problems (test critical threshold ≥ 50%). Finally, Pearson’s correlation analysis (Table 1) indicated that Mindful Attitude was positively correlated with Adult Attachment—Close (*r* = 0.36; *p* < 0.01) and Adult Attachment—Depend (*r* = 0.30; *p* < 0.01) and negatively with Adult Attachment—Anxiety (*r* = −0.44; *p* < 0.01), and FoMO (*r* = −0.30; *p* < 0.01). The latter was also found to be negatively correlated with age (*r* = −0.18; *p* < 0.01), education (*r* = −0.16; *p* < 0.01), Adult Attachment—Close (*r* = −0.18; *p* < 0.01) and positively correlated with Adult Attachment—Anxiety (*r* = 0.32; *p* < 0.01). Notably, the correlations of gender and experience in mindfulness with the other study variables were computed using Spearman’s correlation. The results indicated no significant correlations of gender with Adult Attachment—Close (*r* = 0.12, *p* > 0.05), Adult Attachment—Depend (*r* = 0.07, *p* > 0.05), Adult Attachment—Anxiety (*r* = −0.10, *p* > 0.05), Mindful Attitude (*r* = 0.01, *p* > 0.05), and FoMO (*r* = −0.05, *p* > 0.05). In addition, correlation analysis showed no significant correlations of experience in mindfulness with Adult Attachment—Close (*r* = 0.11, *p* > 0.05), Adult Attachment—Depend (*r* = 0.12, *p* > 0.05), Adult Attachment—Anxiety (*r* = −0.10, *p* > 0.05), Mindful Attitude (*r* = 0.02, *p* > 0.05), and FoMO (*r* = 0.02, *p* > 0.05).

**Figure 1:**
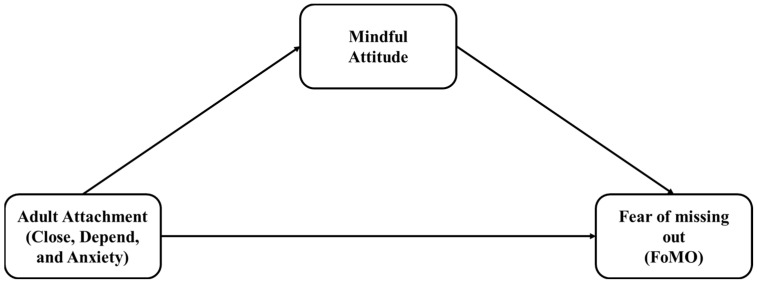
The theoretical models advanced in the current research.

Three mediation models were advanced. Entered one by one, the three adult attachment styles were set as the independent variables. FoMO was set as the dependent variable, while Mindful Attitude was entered as the mediator (Figure 1). 

In setting Adult Attachment—Close as the independent variable and additionally controlling for age, gender, education, Adult Attachment—Depend, and Adult Attachment—Anxiety, the results showed that Mindful Attitude mediated the association between Adult Attachment—Close and FoMO (*B* = −0.03, *BootSE* = 0.02, 95% *BootCI* [−0.073, −0.002]). In addition, in setting Adult Attachment—Depend as the independent variable and controlling for age, gender, education, Adult Attachment—Close, and Adult Attachment Anxiety, the results indicated that Mindful Attitude mediated the association between Adult Attachment—Depend and FoMO (*B* = −0.04, *BootSE* = 0.02, 95% *BootCI* [−0.077, −0.003]). Finally, in setting Adult Attachment—Anxiety as the independent variable and controlling for age, gender, education, Adult Attachment—Close, and Adult Attachment—Depend, the results revealed that Mindful Attitude mediated the interplay between Adult Attachment—Anxiety and FoMO (*B* = 0.03, *BootSE* = 0.02, 95% *BootCI* [0.005, 0.071]). Table 2 reports a summary of the mediation analyses performed in the current research. 

## 4. Discussion

The current research explored the joint contribution of different individual resources in explaining FoMO. Results indicated that mindful attitudes mediated the association between adult attachment and FoMO when Close, Depend, and Anxiety of attachment were entered as independent variables. 

By investigating each path of the mediation models advanced in the current research, our results showed that Close and Depend were positively associated with mindfulness. These findings align with previous studies showing that secure attachment (which involves high levels of Close and Depend) triggers mindfulness [51]. Specifically, as shown in previous studies, the association between secure attachment and mindful attitudes can be explained considering three main points: (1) both secure attachment and mindful attitudes are likely to develop synchronically based on a significant social figure’s response; (2) they also imply attention to emotional stimuli in social relationships; and (3) both involve the development of appropriate qualities and mechanisms for coping with stress [52,53]. Specifically, secure attachment involves security and attention to the present moment without worrying about rejection or threatening experiences [30]. These competencies seem to trigger mindfulness, promoting several core components of a mindful attitude, such as compassion, acceptance, awareness, and present focus [42,51,54,55,56]. In addition, neurological studies suggested that secure attachment and mindfulness report similar neural pathways implicated in modulating individuals’ affect, including response flexibility, self-regulation, and emotional balance [57]. 

Furthermore, according to past research [28], our results revealed that anxious attachment is critical in weakening mindful attitudes. Specifically, Shaver et al. [28] reported that the anxiety and avoidance dimensions of adult attachment significantly predicted lower levels of mindfulness as measured by the Five Facet Mindfulness Questionnaire (FFMQ; [58]). This mechanism can be explained by considering three main points. First, anxious attachment brings low levels of mindful attitude, making individuals hypervigilant to threat-related cues, especially those of rejection and abandonment [27]. In addition, anxious attachment inhibits openness to new information and results in seeking to avoid schema-congruent processing [59]. Yet, while anxious attachment lies in intolerance of ambiguity [15], mindfulness relies on greater cognitive flexibility and openness [59]. This evidence implies that a mindful attitude counters the hyperactivating patterns associated with anxious attachment by promoting adaptive cognitive and emotional skills. 

Finally, our results revealed a negative association between mindful attitudes and FoMO, suggesting that the individual disposition toward high engagement in the present moment and awareness of emotion [9,60] allows individuals to be less affected by negative and dysfunctional affect [61]. This means that a mindful attitude, through re-establishing a sense of control and efficacy and increasing the consciousness of feelings and thoughts, enables the management of the fear and worry of missing significant social events.

### 4.1. Limitations

Despite the evidence described above, the current research showed some limitations and future directions. The correlational design of this research does not allow us to establish a causal relationship between the variables examined but only the presence and nature of the associations between the study variables. Future research should confirm the results of our study, adopting a longitudinal design. Additionally, even though we enrolled 192 subjects, our sample consisted of 78.6% females, and therefore, future studies should consider a more balanced sample when considering the main determinants of FoMO. In addition, we explored Adult Attachment through three styles, namely Depend, Close, and Anxiety. Notably, even though people could show some features of the three styles, only one of them could be predominant [62]. However, we did not consider this aspect in the mediation analysis. Therefore, future studies could investigate the predominance of attachment styles and their further consequences on FoMO. Finally, this research addressed only some intrapersonal factors involved in FoMO (i.e., adult attachment and mindful attitudes). Future research should extend our results, including not only intrapersonal factors such as personality traits, cognitive processes, styles, and strategies, but also cultural and contextual differences [63,64,65,66].

### 4.2. Theoretical and Practical Implications

Finally, our study provided some theoretical and practical implications. From a theoretical point of view, this research extended the growing literature on the central mechanisms related to negative affect, providing a step forward in understanding the relationships between adult attachment and FoMO. In addition, this study provides evidence of the role of a mindful attitude in mediating the association between adult attachment and FoMO, leaving room for the potential role of mindfulness as a protective mechanism against FoMO. From a practical point of view, this latter evidence provided by our study can form a basis for possible research and interventions to promote wellbeing and positive youth development in both adolescents and young adults [67]. Furthermore, the data obtained from this work open novel perspectives for further experimental studies in which mindfulness could represent a protective factor against FoMO. In detail, even though a mindful attitude is relatively stable across the lifespan, improving individual states of mindfulness through specific mindfulness-based interventions could be a useful tool for reducing negative affect, including FoMO. In addition, given that FoMO is closely associated with a wide range of compulsive and unregulated behaviors, such as problematic use of the internet, smartphones, and social media (e.g., [60]), cultivating mindfulness could be helpful in countering dysfunctional practices. 

## 5. Conclusions

In conclusion, our research highlighted the relationship between adult attachment and FoMO via mindful attitudes. Specifically, the results indicated that adult attachment significantly impacts mindful attitudes, which predicts FoMO. In other words, these findings support the view that a mindful attitude is critical in the interplay between adult social relationships and FoMO. Also, our findings support the notion that cultivating mindfulness could be an effective strategy for reducing the adverse effects of FoMO with positive implications for individuals’ psychological and physical wellbeing [4,68,69,70]. Indeed, mindfulness through awareness, self-regulation of attention, and orientation to one’s present experience could weaken maladaptive thoughts and negative emotions, enhancing people’s positive experiences in life [4]. This evidence implies that mindfulness could be critical in regulating distressing thoughts and feelings, ensuring a positive life experience [71,72,73]. Notably, FoMO was found to be a key determinant of compulsive and unregulated social media use [74]. In this vein, our results suggest that mindfulness may be critical in hampering the unpleasant consequences of FoMO in terms of compulsive and unregulated practices associated with social media usage. In summary, the results of the current study can help to enhance the scientific understanding of FoMO, its negative impacts, and the relevance of mindfulness for people’s wellbeing.

## Figures and Tables

**Table 1:** Descriptive statistics and correlations among study variables.

	M	SD	1.	2.	3.	4.	5.	6.	7.
1. Age	23.24	4.33	1						
2. Education (years)	14.11	1.90	0.52 **	1					
3. Adult Attachment—Close	3.36	0.84	0.04	0.11	1				
4. Adult Attachment—Depend	2.67	0.77	0.11	0.17 **	0.45 **	1			
5. Adult Attachment—Anxiety	3.03	1.12	−0.19 *	−0.16 **	−0.46 **	−0.58 **	1		
6. Mindful Attitude	10.26	2.00	0.17 *	0.19 **	0.36 **	0.30 **	−0.44 **	1	
7. FoMO	2.67	0.71	−0.18 **	−0.16 **	−0.18 *	−0.04	0.32 **	−0.30 **	1
α					0.77	0.67	0.90	0.79	0.80

Note. N = 192, gender (0 = F; 1 = M) and past mindfulness experience (0 = no; 1 = yes) were dummy coded; FoMO = Fear of Missing Out. * *p* < 0.05 (two tailed); ** *p* < 0.01 (two tailed).

**Table 2:** A summary of the mediation models performed in the current research.

Independent Variable	Mediator	Dependent Variable	Path a ^1^	Path b ^2^	Direct Effect ^3^	Indirect Effect ^4^	Total Effect
Adult Attachment—Close	Mindful Attitude	FoMO	0.51 [0.154, 0.862]	−0.07 [−0.119, −0.011]	−0.05 [−0.188, −0.079]	−0.03 [−0.073, −0.002]	−0.09 [−0.220, 0.044]
Adult Attachment—Depend	Mindful Attitude	FoMO	0.46 [0.186, 0.731]	−0.08 [−0.147, −0.008]	0.05 [−0.082, 0.185]	−0.04 [−0.077, −0.003]	0.02 [−0.116, 0.147]
Adult Attachment—Anxiety	Mindful Attitude	FoMO	−0.55 [−0.845, −0.262]	−0.07 [−0.119, −0.011]	0.21 [0.099, 0.322]	0.03 [0.005, 0.071]	0.25 [0.138, 0.356]

Note. N = 192. The table shows the path coefficients of the mediation models of the current research. The lower limits and the upper limits of the 95% CI are in the square brackets. FoMO = Fear of missing out. ^1^ The effect of the independent variable on the mediator; ^2^ the effect of the mediator on the dependent variable; ^3^ the effect of the independent variable on the dependent variable while controlling for the mediator; and ^4^ the effect of the independent variable on the dependent variable through the mediator.

## Data Availability

The data presented in this study are available on request from the corresponding author.
